# Poultry Production and Sustainability in Developing Countries under the COVID-19 Crisis: Lessons Learned

**DOI:** 10.3390/ani12050644

**Published:** 2022-03-03

**Authors:** Youssef A. Attia, Md. Tanvir Rahman, Md. Jannat Hossain, Shereen Basiouni, Asmaa F. Khafaga, Awad A. Shehata, Hafez M. Hafez

**Affiliations:** 1 Department of Agriculture, Faculty of Environmental Sciences, King Abdulaziz University, P.O. Box 80208, Jeddah 21589, Saudi Arabia; yaattia@kau.edu.sa; 2 The Strategic Center to Kingdom Vision Realization, King Abdulaziz University, P.O. Box 80200, Jeddah 21589, Saudi Arabia; 3 Department of Microbiology and Hygiene, Faculty of Veterinary Science, Bangladesh Agricultural University, Mymensingh 2202, Bangladesh; tanvirahman@bau.edu.bd; 4 Department of Microbiology and Public Health, Faculty of Veterinary, Animal and Biomedical Sciences, Khulna Agricultural University, Khulna 9100, Bangladesh; jannat@kau.edu.bd; 5 Clinical Pathology Department, Faculty of Veterinary Medicine, Benha University, Moshtohor, Toukh 13736, Egypt; shereenbh@yahoo.com; 6 Pathology Department, Faculty of Veterinary Medicine, Alexandria University, Edfina 22758, Egypt; asmaa.khafaga@alexu.edu.eg; 7 Birds and Rabbit Medicine Department, Faculty of Veterinary Medicine, University of Sadat City, Sadat City 32897, Egypt; awad.shehata@pernaturam.de; 8 Research and Development Section, PerNaturam GmbH, 56290 Gödenroth, Germany; 9 Institute of Poultry Diseases, Faculty of Veterinary Medicine, Free University of Berlin, 14163 Berlin, Germany

**Keywords:** COVID-19, smallholder poultry, sustain ability, poultry supply chain, developing countries

## Abstract

**Simple Summary:**

Globally, poultry production provides high-quality, affordable animal protein, a high chance for investment, job opportunities, and a source of income for smallholders worldwide. However, the recent COVID-19 affected the sustainability of various animal production sectors worldwide, and these influences are more severe in developing countries. The unprecedented negative influences are attributed to the lockdown, movement restriction, and close of some markets. The present review focuses on the impacts of the COVID-19 crisis on poultry production in developing countries in terms of causes and possible solutions to decrease and improve profits. Work must be coordinated between the public and private sectors to facilitate the restoration of the poultry industry to its economic and social position to remedy these problems.

**Abstract:**

Poultry farming is a significant source of revenue generation for small farmers in developing countries. It plays a vital role in fulfilling the daily protein requirements of humans through meat and eggs consumption. The recently emerged pandemic Coronavirus Disease-19 (COVID-19) impacts the poultry production sector. Although the whole world is affected, these impacts may be more severe in developing countries due to their dependency on exporting necessary supplies such as feed, vaccines, drugs, and utensils. In this review, we have discussed poultry production in developing countries under the COVID-19 crisis and measures to regain the loss in the poultry industries. Generally, due to the lockdown, trade limitations have negatively impacted poultry industries, which might exacerbate global poverty. Coordinated activities have to be taken at the private and government levels to arrange soft loans so that these farms can restore their production and marketing to normal levels. In addition, here, we have focused on the supply of farm input, feed, other raw materials, management system, improved breeding efficiency, veterinary services, and marketing of egg and meat, which have to be ensured to secure a sustainable poultry production chain.

## 1. Introduction

In November 2019, the Coronavirus Disease 2019 (COVID-19), caused by Severe Acute Respiratory Syndrome Coronavirus 2 (SARS-CoV-2), which is a highly infectious disease, originated from Wuhan, China, resulting in severe health implications. To date, a total of 5,824,622 deaths and 410,022,236 COVID-19 cases have been reported. Due to the implementation of lockdowns and trade limitations as control strategies, considerable collateral health damage [1] and unprecedented economic crisis have been reported [1,2,3], including the agricultural value chain, such as the livestock production sector, especially in developing countries. Thus, the negative impacts might have more devastating consequences on the most vulnerable people and poorest nations. In this review, we have discussed the effects of the COVID-19 pandemic on poultry production, along with measures that need to be implemented to ensure sustainability in developing countries.

## 2. Impacts of COVID-19 on Food Security and Poultry Production

**Figure 1:**
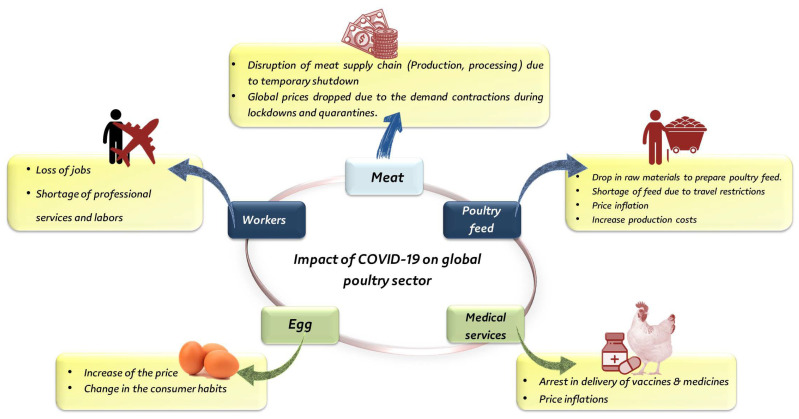
Impact of the COVID-19 pandemic on the global poultry sector.

The COVID-19 pandemic, unlike previous pandemics such as SARA-CoV and Ebola, severely impacted the food supply chain indirectly through disruptions of the downstream stages such as transport and logistics [4,5,6,7,8]. It affected all dimensions of food security, including availability, access, utilization, stability, and sustainability, depending on the industry, the locality, and the financial status of the affected region [9,10]. Figure 1 illustrates the impacts of the COVID-19 pandemic on poultry production.

Generally, animal production sectors in several countries, such as China, the US, the UK, Germany, Spain, Italy, France, and India, were significantly influenced by COVID-19 [4,11,12]; both food availability and demand were also affected [13]. In Canada, the COVID-19 pandemic has damaged the poultry and dairy sectors [14]. This damage even affected the main factors in the industry and animal husbandry, including disruptions in supply chains, scarcity of human resources, the malfunctioning of livestock markets, price volatility, and changes in consumer shopping behavior [15]. No evidence was found indicating that SARS-CoV-2 can be transmitted to humans via poultry products; thus, poultry are resistant to SARS-CoV-2 [16]. However, the poultry industry is negatively impacted globally. It is estimated that the losses might be greater than those due to the avian influenza pandemic of 2006 [17], as 400 million birds were culled globally in 2006. The negative impacts of COVID-19 on the poultry industry are attributed to the indirect effects on necessary supplies such as feed, chicks, medicine and vaccines, and poultry products due to the restriction of movement (Figure 1). Additionally, the strict restrictions during this pandemic disrupted the marketing of poultry products. Due to the closure of markets, farmers were unable to sell eggs at local markets or restaurants, which resulted in substantial financial losses [4]. In developing countries, the negative impacts of COVID-19 on poultry were found to be much higher than those in developed countries because poultry production serves to fight poverty and food scarcity [18]. Table 1 summarizes the negative impacts of SARS-CoV-2 on poultry production observed in some developing countries.

## 3. Plausible Explanations of COVID-19’s Damaging Effects on Animal Production

### 3.1. Concern about the Spread of SARS-CoV-2 from Animals and Social Rumors

At the beginning of this pandemic, the unknown nature of COVID-19 transmission was a major concern, particularly how it spread from animals to humans and vice versa [30,31]. However, poultry are not susceptible to SARS-CoV-2, although they are susceptible to other CoVs such as infectious bronchitis (IBV) that causes respiratory, intestinal, and urogenital problems in chickens [32,33]. IBV has also been reported in pheasants and peafowl [34]. The turkey coronavirus (TCoV) also causes enteric lesions [35,36] and might be involved in the poult enteritis and mortality syndrome (PEMS) [36]. There were also social rumors in some developing countries such as Bangladesh that SARS-CoV-2 can be transmitted via eggs and chickens, leading to price fluctuations for poultry meat and eggs and reducing consumption [31]. It has also been proposed that SARS-CoV-2 can spread through processed foods because it can survive for hours to several days on inanimate surfaces [37]; however, there is no evidence that the virus can be transmitted directly through food, milk, milk products, or eggs [38].

### 3.2. Lockdown and Restrictions of Trades

**Figure 2:**
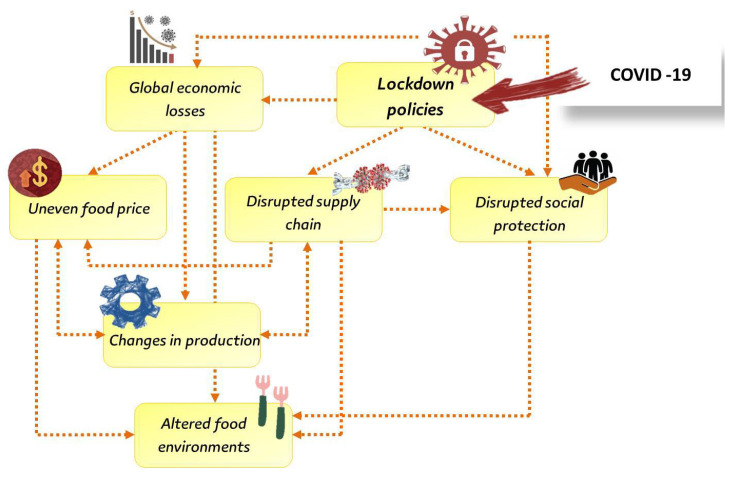
The dynamics of the impacts of lockdown on food security production adapted after HLPE [10]. Several overlapping and reinforcing dynamics affect the food system, such as disruption of the food supply chain and social protection programs, loss of income, shifted food environments, and uneven food prices.

The lockdown of the food distributors, such as food courts, adversely influenced the retail demand. The transport restrictions negatively impacted the products, thus cumulatively affecting the animals in the farms, showing increased animal numbers and high husbandry costs [39,40]. The dynamics of the impacts of lockdown on food security are shown in Figure 2.

The negative impact of the COVID-19 pandemic on the food supply chain can be attributed to the global policies of lockdown according to the One Health world approach, the global economic recession, food price fluctuation, production changes, disrupted social protection, and changes in food environments. This complex relationship between different factors and the disruptions of the food supply chain indicated the slow recovery of the food supply chain in the future and the need for economic reform for faster recovery [20]. The production, imports, and exports of poultry meat in some African countries are shown in Table 2.

Although the poultry production in 2020 was higher than in 2019, there is a significant global decline in import and export supplies, leading to disruptions in the food supply chain [42]. Therefore, there is a trend towards increasing world production, imports, and utilization of poultry meat after COVID-19; however, the exports have declined. For example, Africa showed an increased poultry production and consumption accompanied by decreased imports and exports. In South Africa, poultry production, import, and utilization have decreased, but their exports were not affected. The activities related to poultry production in Africa followed a similar trend to that of the least developed countries. Meanwhile, the least developed countries showed different trends in the production and utilization of poultry products. Moreover, the decrease in exports in Asia may be attributed to the lockdown of borders and ports and/or the trend of the governorates to increase food security and self-sufficiency. The same trend was observed in Africa; however, the values of production, imports, exports, and utilization are significantly different between the two continents, favoring Asia. In North America, all values related to poultry activity have increased; however, in Europe, only the imports have decreased. This indicates that the changes in production, imports, and exports are affected by the stages of development for the world continents.

The negative impacts of lockdown on poultry productions are as follows:

(i) The animal feed supply was disrupted as due to lockdown, several countries such as Argentina and Brazil had to reduce their exports of raw materials of feed such as soybean and corn, which led to a shortage of dry feed in several developing countries [7,8,43]. In several African countries, the cost of chicken feed has increased [44].
(ii) Poultry services were reduced, such as equipment, day-old chicks for stock replacements, feed, vaccines, drugs, diagnostics, and feed additives (vitamins and minerals) [8]. Exporters in several countries also faced a considerable drop in demand for livestock-based foods in major importing markets.
(iii) Market closures and transportation restrictions hampered access to markets and customers, affecting both animals and animal products, such as eggs and meat.
(iv) The shortage of laborers severely impacted the poultry industry in developing countries, since poultry farmers rely on human resources rather than machines [19,20,21].

### 3.3. Small-Scale Poultry Farms

**Figure 3:**
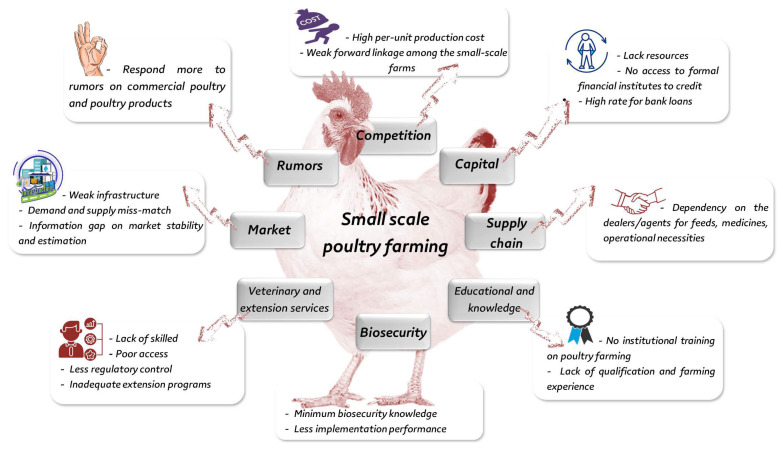
Problems for small-scale poultry farming in developing countries, adapted after [31].

Several small-scale poultry farms are widely distributed in developing countries. Rahman and coworkers summarized the problems that make small farmers more susceptible to any crisis, such as a lack of training on poultry farming, inadequate education and farming expertise, a lack of biosecurity awareness, a lack of infrastructure and marketing system, demand and supply imbalance, information disparity on market stability and estimation, a tendency to respond more to rumors about commercial poultry and poultry products, and a lack of access to veterinary services [19]. The general problems for small-scale poultry farming in developing countries are summarized in Figure 3.

Small-scale poultry production in developing countries is operated informally and represents a high portion of poultry production. In Egypt, for example, small-scale poultry production represents 70% of all poultry production in Egypt [45]. Small-scale production has been found to be severely damaged due to the COVID-19 pandemic since it affected farm management, production systems, and resource endowment. All these effects were, directly and indirectly, related to movement restriction, resulting in less availability of supply of farm raw materials, like feed, essential veterinary services, and manpower, and ultimately production fall. It was reported that most small-scale farmers are on the edge of collapse, facing capital challenges, and are worried that they would be unable to continue their business owing to livestock supply chain disruptions. The severe negative impact on small-scale production in developing countries can be attributed to the following factors [21]:

(i) Lockdown harmed small-scale production severely because small-scale producers in developing countries rely more on labor than machinery.
(ii) Most small-scale-producing farms, including poultry farms, operate informally and, therefore, are excluded from the stimulus plans offered by governments to private businesses. Smallholder poultry producers mostly live in rural areas (sell eggs and live birds and purchase day-old chicks, feed, drugs, and disinfectants) [46]. However, the animal husbandry services were severely interrupted due to lockdown and movement restrictions. Because most small-scale poultry farms are unregistered, they are not eligible for government stimulus programs [47].
(iii) Compared with large-scale production, small farmers earn fewer benefits due to increased production expenses [30,48], and they are reliant on the local dealers for feeds, medications, and operating capital [48,49,50].
(iv) The implementation of sanitary measures in small-scale farms is difficult due to the lack of logistical and financial resources [31], making them more vulnerable to other infections.

Therefore, it is recommended to enhance the preparedness and resilience of small-scale poultry production systems in developing countries to tackle future pandemics.

## 4. Prospective to Improve Poultry Husbandry under COVID-19 Pandemic

Challenges facing poultry sustainability in developing countries, problems, causes, and suggestions are summarized in Table 3.

Creating techniques that will help boost nutrition and food security is essential while simultaneously containing COVID-19 in African countries and handling significant socioeconomic problems [30]. Moreover, these techniques could also be applied to many Asian countries. The global spread of COVID-19 forced the imposition of self-isolation and physical distance to reduce the transmission risk and ensure human safety. The medical facilities and positive human responses were of particular concern; however, there are growing concerns about the effects of the COVID-19 pandemic on the food chain due to the lockdown of borders, seaports, and airports, its consequences on food security, and its influences on the poultry sector [18,51]. In the post-COVID-19 period, the policy of adapting backyard poultry as an alternative source of income not only increases poultry production but also ensures the availability of animal protein to the poorer sections of society, improves their purchasing power, and protects against labor “reverse migration” [17]. Small-scale poultry farmers may play a key role in supplying the demand for animal products in emerging markets. Several initiatives to give marginal farmers chicks, feed, and medicine were launched by the government and private organizations in India, Bangladesh, and Cambodia during the lockdown to reduce the catastrophic effect of COVID-19 on the agricultural economy [31].

The One Health concept integrates human, environmental, and animal health to regulate and prevent disease outbreaks [52,53,54]. The proliferation of the novel coronavirus created great health hazards to both humans and animals at various times, resulting in significant economic losses and environmental damage [4,8,31,51,55]. Chickens, ducks, and pigs are resistant to the SARS-CoV-2 virus or have a low vulnerability to this unique virus. However, many COVID-19 outbreaks have been reported in several countries in association with meat and poultry processing plants [56]. Due to the transmission of infectious agents outside workplaces to family and contacts of workers, the detection of hazard variables particular to meat-packing plants may aid in developing customized control programs in this business, which exemplifies the personal connection between workplace safety and public health.

According to the Centers for Disease Control and Prevention (CDC), 4913 cases with 20 deaths have been reported in different meat and poultry processing units [57]. It is recommended that all employees and supervisors should get regular training on infection control, workplace safety, and health tailored to their reading levels and chosen languages. The training should cover the following points: what employees should do if they feel sick before or at work; COVID-19 symptoms; sick leave regulations; social distance guidelines; proper use of PPE and face shield; sanitation facilities; application for screening when it becomes more readily accessible; possible transmission routes at work and elsewhere. Culturally competent trainers should deliver training in a context social distance is maintained and in the dialects used by employees and pay attention to the different levels of education [58].

When workers in poultry processing plants tested positive for COVID-19, public health officials suspended the plant for ten days. Those who have been identified as cases or close contacts have been told to self-isolate. According to the Canadian Food Inspection Agency (CFIA), there is no evidence that food is a common cause or route of viral transmission. No reports have yet linked the spread of COVID-19 to food or packaged food. As a result, there is no need to recall chicken products distributed by this plant [12]. Meat-packing facilities, abattoirs, and processing plants are at high risk of transmission of COVID-19. They are depicted as major sources of regional epidemics and, on rare occasions, as a critical source of a nationwide epidemic when the disease was otherwise under control. The identified danger to meat and poultry facility operations, part of the national reaction to COVID-19, necessitates immediate action to reduce worker risks and protect facility performance and the food chain. Cooperative management is necessary to minimize workplace hazards, enhance cleanliness and sanitation, and apply operating procedures and source control in poultry processing plants, which may help reduce the incidence of COVID-19 among employees. The following items are recommended during food processing: (1) applying soap and water on both hands for at least 20 s; (2) cleaning and sanitizing surfaces regularly; (3) thoroughly cooking the meat; (4) minimizing the risk of cross-contamination between cooked and raw foods.

## 5. Lessons Learned from the COVID-19 Pandemic

The COVID-19 pandemic has shown us how vulnerable we are as human beings to the invisible enemy, the SARS-CoV-2. The devastating impact of the virus is not limited to human civilization, as animals, the environment, and the ecosystem are all affected. In addition to the loss of human life, the actual impact of the pandemic on the global economy is beyond our estimation. These impacts are more severe in developing countries than those in developed countries. SARS-CoV-2 is zoonotic in nature. The pandemic has taught us to implement more basic research and surveillance on animal pathogens in the wild using the One Health-based approach. Findings of research, such as One Health-based research, will help in adopting intervention strategies for reducing contact or spread of infectious by creating a buffer zone. The factors that trigger the virus to cross the species barrier need to be revealed to better control future pandemic outbreaks. Besides, to overcome the impacts on the animal sector, such as poultry production and supply chain, attention has to be paid to smart farm management, including feed supply, biosecurity, veterinary service, human resources, and product sales. Moreover, it is now time to change relevant government policies so that farmers and farm owners who suspended their work due to lack of the necessary supplies and/labor receive compensations smoothly as soon as possible. Other measures are also recommended to implement the One Health concept (Table 3). Collectively, the following measures are described by several authors [16,59,60]:

(i) Application of biosecurity in animal farms and implementation of suitable hygienic measures
(ii) Assessment of the economic impacts of COVID-19 on animal production sectors.
(iii) Assessment of the social effects of COVID-19 on individuals and development of more effective anti-SARS-CoV-2 medications and vaccines and diagnostics.
(iv) Educating the public about the spread of SARS-CoV-2.
(v) Cooperation between the different agencies and using veterinarians’ and physicians’ experiences in viral isolation programs and disinfection under the supervision of health authorities
(vi) Regular risk assessment of healthcare and nonhealthcare workplaces to ensure safe work practices.
(vii) Identifying potential reservoirs and analyzing risk factors and surveillance of live animal marketplaces and humans in touch with animals.
(viii) Devising strategies and collecting funds for the implementation of preventative and control measures.

Moreover, the One Health strategy focuses not only on the pathogens and their host but also on the ecology and environment where the pathogens are maintained.

Thus, anthropogenic activities, such as urbanization, agricultural expansion, deforestation, and globalization, that destroy the ecosystem may cause the emergence of pathogens. Findings of the One Health-based research will help adopt intervention strategies for reducing contact or spread of infectious by creating a buffer zone.

## 6. Conclusions

The COVID-19 pandemic has significantly reduced poultry production and supply chain at the local and international levels, resulting in severe economic loss. In this pandemic situation, we have to take measures to ensure sustainable poultry production for regaining the loss in poultry industries. Such loss can be overcome if sustainable poultry production is ensured at local and industry levels. Compensating the farmers is an important step to retaining their production system. Continuous supply of raw materials in terms of day-old chicks, feed, vaccines, and human resources and a better farm management system are necessary to maintain sustainable poultry production during the COVID-19 era.

## Figures and Tables

**Table 1:** Impacts of COVID-19 on poultry production in some developing countries.

Name of Country	Adverse Effects on Poultry Production	References
Bangladesh	Disruption of necessary supplies such as feed, vaccines, medicines, and equipment People are unwilling to consume chicken and poultry products due to rumors that SARS-CoV-2 is transmitted through poultry and poultry products Discrepancy between supply and demand The poultry sector lost $825 million due to this pandemic The poultry feed segment alone lost $98 million from January to May 2021	[19]
Egypt	Negatively impacted many phases of the poultry logistic system, including production, transport, processing, marketing, retailing, and consumption Farm profit fell by 20% in the first half of 2020, compared with 2019 Disruption of the production inputs (e.g., breeds, feeds, drugs, and vaccines)	[20,21,22]
India	Dramatic negative impacts on the poultry industry Projected losses were estimated to be $3053 million The economic impact is not consistent across the country due to geographical variations in consumption patterns	[23]
Indonesia	Disruption of the supply chain system The market price of chicken dropped dramatically Economic growth fell from 4.97% to 2.97% A significant drop in demand for broiler chickens	[24,25]
Ghana	Decrease in the importation of animals and livestock products Reduction in the availability of feed resources and farm inputs for animal production, leading to increase of price Animal production activities, including feeding, management, and disease control adversely affected as a result of the lockdown	[26]
Myanmar	Negative impacts on chicken and egg industries A decrease in demand in 60% broiler farms 40% of layer farms Approximately 30% of broiler farms and 10% of layer farms have closed 42% of long-term farm workers have been laid off Reduction of broiler and egg prices	[27,28]
Nigeria	A substantial drop in sales and market price of eggs (dropped approximately 20%)	[29]
Saudi Arabia	The COVID-19 pandemic affected poultry consumption, transportation, and poultry business	[18]

**Table 2:** Production, imports, exports, and utilization of poultry meat (thousand tonnes-carcass weight equivalent) in the world continents from 2019 to 2020 (before and during the COVID-19 pandemic).

Continents of the World	Production		Imports		Exports		Utilization	
	2019	2020	2019	2020	2019	2020	2019	2020
World	131,562	133,266	12,451	12,501	14,241	14,226	129,754	131,596
Europe	22,089	22,289	1299	1156	2622	2675	20,759	20,765
Asia	49,669	50,367	6476	6836	2871	2739	53,309	54,475
Africa	6568	6758	1962	1872	117	114	8412	8516
South Africa	1816	1965	540	486	57	57	2299	2394
Central America and the Caribbean	5279	5399	1873	1804	31	42	7121	7162
South America	22,030	22,263	377	356	4588	4498	17,820	18,121
North America	24,361	24,592	344	370	3932	4086	20,728	20,924
Low-income food-deficit countries	7645	7351	1407	1329	58	50	8995	8630
Least developed countries	3572	3656	1170	1108	21	17	4722	4748

Source: FAO, 2021, [41].

**Table 3:** Sustainability of poultry production in developing countries, problems, causes, and suggestions.

Facing Sustainability Problems	Reasons/Causes	Suggestions
Disruption of oultry supply chain	Persistence of the pandemic, rumors, panic, blockage of transportation restrictions, delivery delays, sales losses, operational disruptions, price fluctuation, lockdown, blockage of distribution channel, imbalance between supply and demand, and consumer’s dilemma due to COVID-19 transmission	Raise public awareness Withdrawal of transport restrictions Collaboration among governments, nongovernmental organizations, veterinarians, and other health professionals Hygienic application of poultry operation Supporting small and medium farmers and proper market mentoring Searching for alternative feed sources, use of phytogenics, and shift of dietary lifestyle Coordination among farmers, input suppliers, veterinary professionals and nutritionists, poultry associations, and governments Proactive measures from the government to improve the welfare of breeders and farmers
Price fluctuations for poultry meat and eggs	Social rumors that SARS-CoV-2 is transmitted by eggs and chickens	Educational program about SARS-CoV-2 transmission No evidence that poultry and poultry products play a role in the transmission of the virus
COVID-19 infects the employees of chicken processing plants	Lack of awareness and community guidelines, crowded conditions, and vaccination hesitation	Breaks and shifts with different start and stop times Vaccination of workers Hygienic measures such as frequent hand washing and disinfection Personal protective equipment Regular monitoring of workers Implementation of social distancing Disinfection of equipment and surfaces Restriction of human access
Disruption of meat supply chain	Disruption of meat production and processing and temporary shutdown of hotels and restaurants	Opening the poultry production and processing sites, hotel, and restaurants following COVID-19 health guidelines Government support of small commercial farmers financially
Low capital of the farmers, education and knowledge gap, lack of biosecurity and management training of the workers, and propaganda	Reducing poultry consumption, transportation, cost of poultry farming, consumer trust, and product quality	Low-interest credit from financial institutions Developing farmer’s knowledge and skills Building awareness for biosecurity guidelines
Impact on chicken and egg production, low intake of proteins, and loss of employment	Temporary or permanent closure of poultry farms, low demand, and cash flow problems	Government and NGOs should come forward to support small-scale poultry farmers
Effects on animal production, lower consumption of proteins, and high market price	Restrictions on global travel, flaws in the feed, veterinary, insurance, and other supply networks	Various governments and stakeholders should come forward to reduce the adverse effects on the livestock sector

## Data Availability

Not applicable.
